# Recombination Rate and Selection Strength in HIV Intra-patient Evolution

**DOI:** 10.1371/journal.pcbi.1000660

**Published:** 2010-01-29

**Authors:** Richard A. Neher, Thomas Leitner

**Affiliations:** 1 Kavli Institute for Theoretical Physics, University of California, Santa Barbara, California, United States of America; 2 Theoretical Biology and Biophysics, Los Alamos National Laboratory, Los Alamos, New Mexico, United States of America; Imperial College London, United Kingdom

## Abstract

The evolutionary dynamics of HIV during the chronic phase of infection is driven by the host immune response and by selective pressures exerted through drug treatment. To understand and model the evolution of HIV quantitatively, the parameters governing genetic diversification and the strength of selection need to be known. While mutation rates can be measured in single replication cycles, the relevant effective recombination rate depends on the probability of coinfection of a cell with more than one virus and can only be inferred from population data. However, most population genetic estimators for recombination rates assume absence of selection and are hence of limited applicability to HIV, since positive and purifying selection are important in HIV evolution. Yet, little is known about the distribution of selection differentials between individual viruses and the impact of single polymorphisms on viral fitness. Here, we estimate the rate of recombination and the distribution of selection coefficients from time series sequence data tracking the evolution of HIV within single patients. By examining temporal changes in the genetic composition of the population, we estimate the effective recombination to be *ρ* = 1.4±0.6×10^−5^ recombinations per site and generation. Furthermore, we provide evidence that the selection coefficients of at least 15% of the observed non-synonymous polymorphisms exceed 0.8% per generation. These results provide a basis for a more detailed understanding of the evolution of HIV. A particularly interesting case is evolution in response to drug treatment, where recombination can facilitate the rapid acquisition of multiple resistance mutations. With the methods developed here, more precise and more detailed studies will be possible as soon as data with higher time resolution and greater sample sizes are available.

## Author Summary

Evolution, in viruses and other organisms, is the result of random genetic diversification by mutation or recombination and selection for survival. In most organisms, evolution is too slow to be observed directly and the evolutionary past has to be reconstructed from static snapshots of the population. This reconstruction requires simple models of evolution that typically neglect selection or recombination. In vigorously evolving organisms like HIV, such assumptions are questionable. However, HIV evolves rapidly enough that substantial evolution is observable during a chronic HIV infection within single patients. Using such time series data of evolution, we estimate the effective recombination rate of HIV (the rate of viral sex) to be similar to the mutation rate, rather than much larger as previously reported. We also study the strength of selection exerted on the virus by the immune system. We find that about 15% of the observed virus variants with mutations in the surface protein are favored and selected at a rate of 0.8% to 2% per virus replication cycle. Knowledge of the recombination rate and the strength of selection is essential for quantitative modeling and understanding of HIV evolution.

## Introduction

The human immunodeficiency virus (HIV-1) ranks among the most rapidly evolving entities known [1], enabling the virus to continually escape the immune system. After infection with HIV, patients typically enter an asymptomatic period lasting several years during which the virus is present at low to medium levels, typically at a viral load of 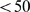 to 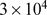 copies per ml plasma. Nevertheless, the number of virions produced and removed is estimated to be around 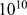 per day with a generation time slightly less than two days [2]. Due to this rapid turnover and the high mutation rate of 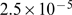 per site and generation, the sequence diversity of HIV within a single patient can rise to 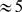
% ( *env* gene) within a few years and the divergence from the founder strain increases by 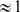
% per year [3], although this rate is not constant [4]. The genotypic diversity is subject to positive selection for novel variants that are not recognized by the host immune system or that reduce the sensitivity to anti-retroviral drugs [5]–[7], as well as to purifying selection by functional constraints [8]. In addition to high substitution rates and strong selection, genomes of different HIV particles within the same host frequently exchange genetic information. This form of viral recombination works as follows: Whenever a cell is coinfected by two or more viruses, the daughter virions can contain two RNA strands from different viruses[9],[10]. In the next round of infection, recombinant genomes are generated by template switching of the reverse transcriptase while producing cDNA. It has been shown that recombination in HIV contributes significantly to the genetic diversity within a patient [11]–[13]. In cases of super-infection with several HIV-1 subtypes, recombination can give rise to novel forms that become part of the global epidemic [14].

The observation of recombinant viruses after a change in anti-retroviral drug therapy [15] suggests that recombination might play an important role in the evolution of drug resistance, as predicted by theoretical models [16]. The amount by which recombination speeds up the evolution of drug resistance depends on the parameters governing the population dynamics [17], many of which are not known to sufficient accuracy. *In vitro* estimates of the recombination rate have shown that the reverse transcriptase switches templates about 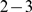 times while transcribing the entire genome, resulting in a recombination rate of 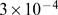 per site and generation [18],[19]. However, the bare template switching rate is only of secondary importance, since recombination can generate diversity only if the virion contains two RNA strands that originate from different viruses, which requires coinfection of host cells[20]. The effective *in vivo* recombination rate is therefore a compound quantity, to which the template switching rate and the probability of coinfection of a single host cell contribute. This effective recombination rate has been estimated with coalescent based methods developed in population genetics [13],[21]. These methods use a single sample of sequences obtained from the diverse population and estimate the recombination rate from topological incongruencies in the phylogenetic tree of the sequence sample. Together with an estimate of the mutation rate, this allows to estimate the recombination rate in real time units. Shriner et al. [13] report an estimate of 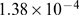 per site and generation, implying almost ubiquitous coinfection of host cells.

Here, we present a different method to estimate recombination rates from longitudinal sequence data, which has been obtained from 11 patients at approximately 6 month intervals [3],[22]. By comparing sequence samples from successive time points, we can estimate recombination rates from the distance and time dependence of the probability of cross-over between pairs of polymorphic sites. We find that the effective rate of recombination is 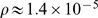 per site and generation. Furthermore, we estimate the strength of selection on nonsynonymous polymorphisms by measuring the rate at which allele frequencies change. We find that a fraction of about 15% of the observed nonsynonymous polymorphisms are selected stronger than 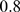
% per generation.

## Results

Time series sequence data of the C2-V5 region of the *env* gene was available from eleven patients over a time-span of 6-13 years with sequence samples taken typically every 6–10 month [3],[23]. At each time point, about 5–20 viruses had been sequenced successfully. These sequences were aligned as described in Methods. We will use the temporal variation in genetic diversity to estimate the recombination rate and selection coefficients that govern the dynamics of the viral population.

### Recombination rate

**Figure 1:**
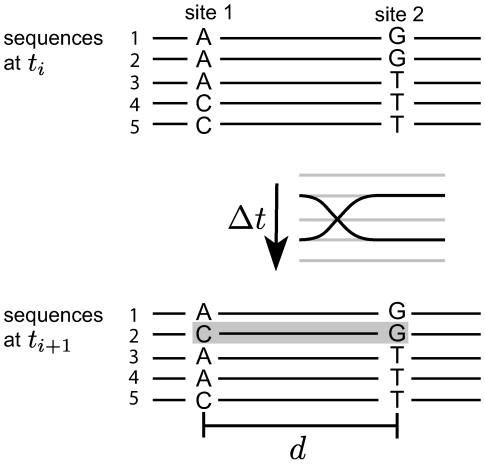
Recombination produces new haplotypes. At time point 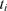, a pair of polymorphic sites is observed in three different combinations A…G, A…T, and C…T. Recombination can generate the missing haplotype C…G (grey box) from one time point to the next with a rate proportional to the product of the distance 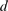 between the two sites and the time interval 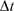 between the two observations. Since no other process depends on this combination of parameters, we can estimate the recombination rate using this dependence.

Recombination produces new combinations of alleles from existing genetic variation and randomizes the distribution of genotypes. To illustrate this process and the challenges of estimating recombination rates, consider the pair of polymorphic sites in Figure 1. Generically such a pair will have arisen by the following sequence of events: (i) Site 1 becomes polymorphic by mutation, e.g. A 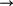 C. (ii) A mutation occurs at site 2 on a genome that carries one of the variants of site 1, e.g. giving rise to the haplotypes A…T, A…G and C…T. (iii) The missing haplotype, in this example C…G, can be generated by further mutation (A 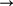 C at site 1 or T 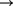 G at site 2) or by crossing over two of the existing haplotypes, as illustrated in Figure 1. In population genetics, the occurrence of the fourth haplotype is often taken as sufficient condition for recombination (the four gamete test[24]). While this is true for bacteria and eukaryotes because of their low mutation rates, the HIV population within a patient is large and mutates rapidly [25]. Hence, the biggest challenge in estimating recombination rates is to separate recombination from recurrent mutations or homoplasy. A second confounding effect stems from the small number of sequences available per time point, such that the sequences containing the fourth haplotype could have been missed due to undersampling. To disentangle these two effects from recombination, we make use of the fact that only the recombination rate depends strongly on the distance between the two sites, while recurrent mutations and sampling noise should not.

**Figure 2:**
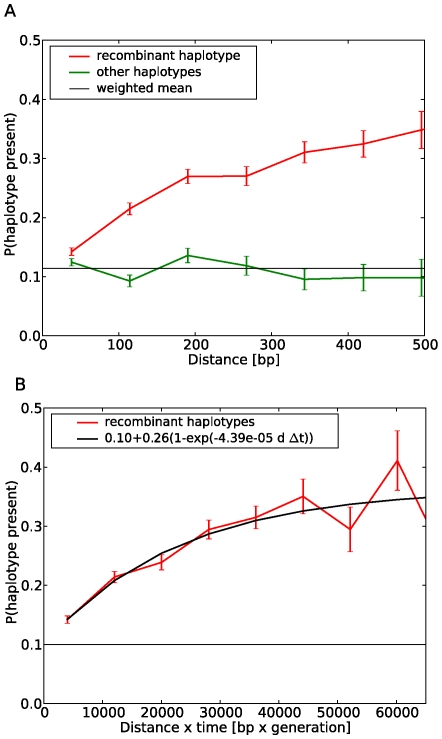
Estimating recombination rates from time resolved data. Panel A shows the probability of finding a haplotype that is not detected at time 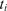 in the sample at 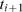 as a function of the separation 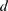 of the sites. The data labeled ‘recombinant haplotypes’ refers to those combinations, that can be generated by recombination from the alleles detected at time 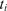 and displays a pronounced distance dependence. The data labeled ‘other haplotypes’ refers to pairs containing at least one allele not detected at time 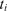, implying an additional mutation or undersampling. The data is averaged over all time points, all patients, and those pairs of polymorphic sites, where both alleles at both sites are seen at least 3 times. Panel B shows the probability of finding the missing haplotype as a function of the product of distance 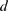 and time interval 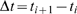. The fit to the data is shown in black with fit parameters indicated in the legend.

For each pair of biallic sites at time 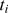 that was found in three of the four possible haplotypes, we asked whether the missing haplotype is observed the at time 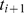 (comp. Figure 1) and calculated the frequency of this event as a function of the separation of the two sites, as shown in Figure 2A. This frequency increases with the separation of the two sites from about 0.1 to about 0.35 at 500 bp separation, in line with the expectation that recombination is more rapid between distant sites. To corroborate that this distance dependence is indeed due to recombination, we performed the following similar analysis: The curve labelled “other haplotypes” in Figure 2A shows the frequency of observing a haplotype at time 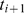, which contains alleles not observed at time 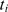, again averaged over all available data. Any such haplotype could have arisen by mutation in the time interval between 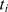 and 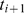, or could have been present at time 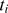 but not sampled. It cannot, however, be assembled by recombination from the alleles found at time 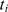. The important observation is that the frequency of observing such a haplotype does not increase with distance. This is consistent with our expectation that an additional mutation or undersampling should be independent of an additional polymorphism nearby. The clear separation between the two classes of haplotypes suggests that the contribution from homoplasy and sampling can be accounted for by a distance independent constant.

**Figure 3:**
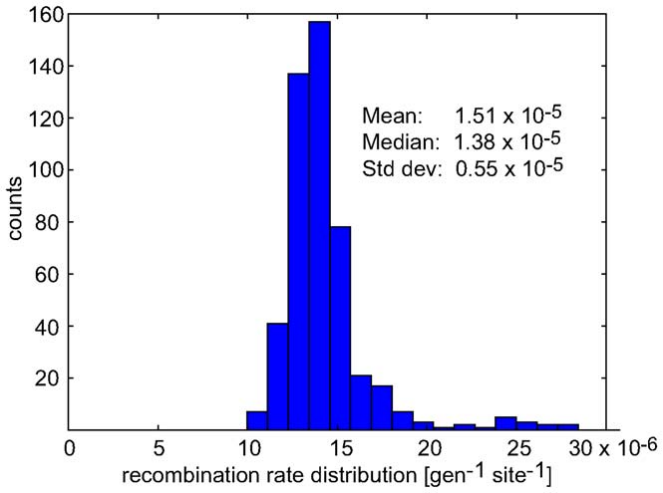
Bootstrap distribution of the recombination rate estimate. The variability of the recombination rate estimator was assessed by repeating the fit 500 times with data of eleven patients sampled with replacement from the original eleven patients. The mean, median, and standard deviation of the distribution are given in the figure.

The probability of recombination between two sites increases with the product of the time elapsed and the distance between the sites, rather than with distance alone as plotted in Figure 2A. Panel B of Figure 2 shows the probability of appearance of a putative recombinant haplotype as a function of the product of distance and number of generations (generation time 2 days). To estimate the recombination rate, we have to know how the observed saturation behavior is related to recombination. Let 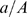 and 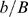 be the alleles at site 1 and 2 and 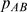 the frequency of the missing haplotype 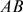. The probability not to detect haplotype 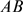 in a sample of size 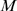 is
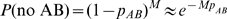
(1)Assuming the allele frequencies remain constant, 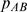 will relax from its initial value 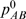 to the product of the allele frequencies 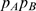 as 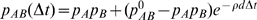 through recombination[26]. The frequency of detecting a haplotype at time 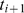, given it was not detected in the previous sample at time 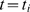, is therefore 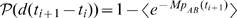. This quantity, averaged over all pairs of polymorphic sites at distance 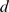 such that 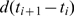 falls into a specified bin, is shown in Figure 2B (the average extends over all patients and time points). To understand how 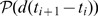 depends on the recombination rate, it is useful to consider the two limiting cases of small and large 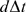


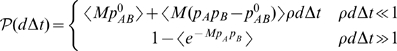
(2)where we assumed that 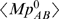 is small compared to one (inspection of Figure 2B shows this is true, we discuss this in more detail in Methods). Hence, the recombination rate is proportional to the slope 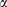 of 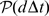 at 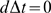. The intercept 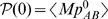 is simply the probability that we detect 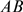 at time 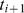 in absence of recombination, given we missed it at time 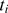. We determine the slope and the intercept by fitting the function 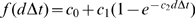 to the data. The recombination rate is then given by
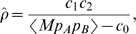
(3)where 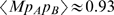 is measured directly. The best fit yields 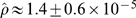 recombinations per site and generation (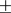 one standard deviation). The uncertainty of 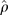 was estimated by resampling the patients with replacement 500 times. This bootstrap distribution of the recombination rate estimate is shown in Figure 3. We have assumed that the allele frequencies 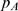 and 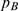 remain constant in the interval 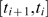. We will see below, however, that some allele frequencies change rapidly. We expect that repeated sweeps will cause our method to overestimate the recombination rate: When the frequencies of the minor alleles increase, the missing haplotype is produced more rapidly then expected.

### Selection

Positive selection on the variable regions in *env* and purifying selection on the conserved regions have been repeatedly reported in the literature [5], [23], [27]–[29]. Most of the these studies compared the rates of synonymous and non-synonymous substitutions (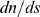 ratio). An observation of 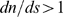 indicates selection for novel variants, while 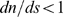 indicates that the amino acid sequence changes more slowly than the nucleotide sequence, indicating functional constraint at the protein level. The overall rate of substitutions, however, yields only very limited information about the strength of selection.

Selection for a specific variant implies that this variant confers an elevated rate of reproduction compared to the population mean. Ignoring random drift, the strength of selection 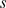 is related to the rate at which the frequency 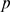 of the variant changes [26]:
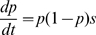
(4)This equation has a straightforward solution 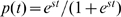 and from two measurements of 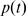 at time 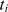 and 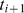, 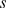 can be determined to be 
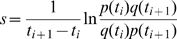
 (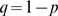)[11] (for a review on selection and drift, see [30]). However, when using this formula, emphasis is put on rare alleles, whose frequencies can't be measured accurately in small samples. It proves more robust to estimate the rate of allele frequency change directly as 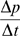, where 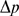 is the difference in allele frequency between consecutive samples and 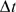 is the time interval. The discrete derivative 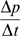 can serve as a proxy for selection which is less sensitive to rare alleles. The observed 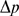 will be a sum of contributions from selection, noise from random sampling, and genetic drift. The latter can be estimated by measuring 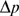 for synonymous polymorphisms which are putatively neutral and their observed frequency changes are assumed to be dominated by sampling noise. However, they can be affected by selection on nearby non-synonymous polymorphisms (hitch-hiking) or be themselves under selection, e.g. for translation efficiency or RNA secondary structure.

**Figure 4:**
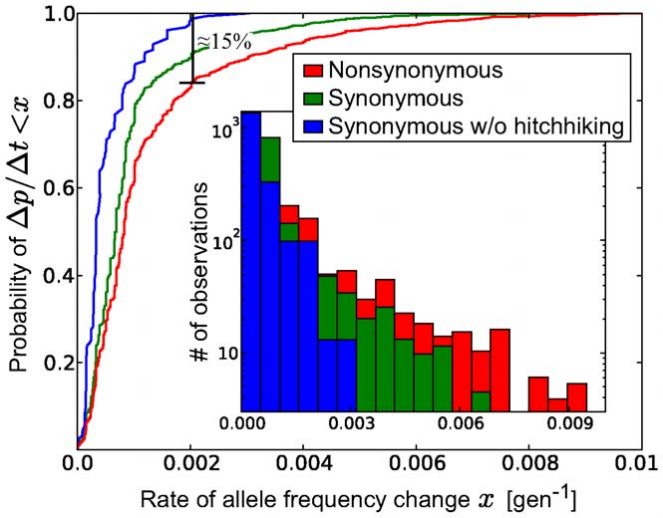
Selection on non-synonymous mutations. The figure shows cumulative distributions of the observed rate of change 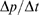 of the allele frequencies 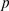 between two consecutive samples at times 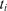 and 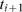 for non-synonymous polymorphisms, synonymous polymorphisms, and synonymous polymorphisms at least 100bp away from the nearest fast changing non-synonymous polymorphism (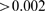/generation), see methods. The inset shows the corresponding histograms on a logarithmic scale. Only pairs of time points with sample sizes greater than 10 sequences are included.

Despite the limited resolution of 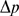 and 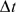 due to small sample sizes and large time intervals between samples (6–10 month), we can make a meaningful statement about the strength of selection when averaging over all sites, patients and time points. Figure 4 shows the cumulative distributions of the rates of change of allele frequencies observed during the interval between two consecutive time points for non-synonymous and synonymous polymorphisms. The histograms are shown as insets in the Figure. There are consistently more fast changing non-synonymous polymorphisms than there are synonymous ones, suggesting that a fraction of the non-synonymous polymorphisms is indeed responding to selection. To check whether the fast changing synonymous polymorphisms can be attributed to hitchhiking, we excluded synonymous polymorphisms that are closer than 100bp to a non-synonymous polymorphism that changes faster than 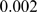 per generation. The resulting distribution is much narrower with no allele frequency changes beyond 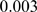 per generation, indicating that the fast changing synonymous polymorphisms are indeed “hitch-hiking”. The cumulative histograms can be compared by the Kolmogorov-Smirnov test, which uses the maximal vertical distance between curves as a test statistics. The test reveals that the non-synonymous distribution is significantly different from both the unconditional synonymous distribution (p-value 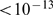) and the synonymous distribution without hitch-hiking (p-value 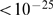). Note that not all observations are independent since nearby sites are linked and move coherently. Hence, realistic p-values will be larger.

The fastest allele frequency changes detected are about 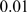 per generation, which is our resolution limit (6 month 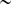
100 generations). This low time resolution results in a tendency to underestimate the rates of change, while the finite sample size will generate spurious frequency changes due to sampling noise. However, the narrow distribution of allele frequency changes of synonymous polymorphisms excluding hitchhiking suggests that the contribution from sampling noise is small (on the order of 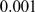 per generation). Hence, the tail of the histogram of the frequency changes of nonsynonymous polymorphisms contains a rough measure of the distribution of selection coefficients[31]: about 15% of the observed non-synonymous polymorphisms change faster than 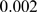 per generation, compared to almost none of the synonymous polymorphisms. Assuming that this difference is due to selection, we conclude that about 15% of the observed non-synonymous polymorphisms are positively selected with 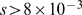 per generation. In the last step, we rearranged Eq. (4) to obtain 
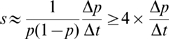
 with 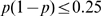. Most of the strongly deleterious polymorphism are of course never observed in the samples.

## Discussion

The dynamics of HIV within a single patient is characterized by large diversity due to high mutation rates, intense selection, frequent recombination and stochasticity resulting from bursts of viruses originating from a single cell. The simultaneous importance of these four evolutionary forces makes HIV evolution difficult to analyze with traditional population genetic methods, which typically assume that one of the evolutionary forces is predominant. Coalescent based methods, for example, assume that evolution is neutral, i.e. stochasticity dominates over selection. While it is possible to include recombination or selection into a coalescent description [32],[33], the analysis becomes difficult and computationally demanding. Estimators based on site frequency spectra, like Tajima's 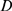, work best when recombination is strong compared to selection. Phylogenetic analysis, on the other hand, assumes absence of recombination. These methods have been designed to infer parameters of the population dynamics from snapshots of the population, which is a formidable challenge. The lack of time series data requires the assumption of a model of the population dynamics, which can be extrapolated back in time to the most recent common ancestor. In neutral models, the time to the most recent common ancestor is on the order of 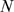 generations, 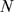 being the population size. During this long time interval, there is ample opportunity for selection or demography to invalidate the assumption of the model.

If time series data is available, the task of estimating parameters is greatly simplified since one can trace the dynamics of alleles and genotypes directly. Such longitudinally sampled data has for example been used to gauge the molecular clock of bacterial evolution [34]. We have used time resolved data from 11 patients to estimate parameters of the population dynamics directly. In accordance with existing studies, we find that recombination in HIV is frequent and contributes significantly to sequence diversity [11]–[13],[21]. The template switching rate of HIV is known to be about 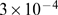 per site[18],[19]. For template switching to result in a novel genetic variant, the two RNA strands in the infecting virus have to be different, which implies co-infection of the cell the virus originated from. Any estimate of recombination rates from sequence diversity will therefore measure an effective recombination rate 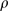 being approximately the product of the coinfection probability, the probability of forming a heterozygote and the template switching rate. Our estimate of this effective recombination rate 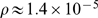 per site and generation is about a factor of 20 lower than the template switching rate, but still implies a probability of coinfection of about 10%. Our estimate is lower than the estimate by Shriner et al. [13], who estimated 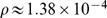 per site and generation. However, two other estimators also reported in that paper yielded lower recombination rates comparable to our estimate.

In our analysis we have assumed that the rate of recombination is constant across the *env* gene. However, the breakpoint distribution in circulating recombinant forms show strong variation along the genome, with particulary little recombination in *env* [35]. The variation of the breakpoint distribution can largely be explained by low sequence homology between different subtypes and dysfunctional recombinants [36]. In the present study, however, all patients were infected with a single subtype and gradually built up diversity which remained much lower than the distance between subtypes. The effects causing recombination rate variation should therefore be of minor importance.

By comparing the distribution of the rate of allele frequency changes of synonymous (putatively neutral) and nonsynonymous (possibly selected) polymorphisms, we estimated the distribution of selection coefficients on single sites. We find that 15% of the observed nonsynonymous polymorphisms are selected with coefficients greater than 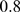
% per generation. In using the distribution of allele frequency changes to infer selection coefficients, we have assumed that each locus is selected for its own effect on fitness, rather than changing its frequency due to selection on a linked neighboring locus [37] or some epistatic combination of loci [38],[39]. A sweeping polymorphism “drags” along neutral variation in a region 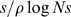 [40], which using our estimates of 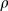, 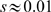 and an effective population size of 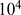 evaluates to 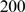
bp. This is consistent with our finding that most of the rapid allele frequency changes of synonymous polymorphisms occur in the vicinity (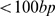) of rapidly changing non-synonymous polymorphisms (Figure 4).

The sequence diversity in our samples is on the order of 3% [3], such that two polymorphisms are expected to be on average 30bp apart. Roughly half of the observed polymorphisms are non-synonymous, of which 15% are under strong positive selection. Hence the distance between simultaneously sweeping loci is on the order of 400bp, which is of the same order as 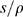. If the rate of sweeps was much higher, sweeps would cease to be independent and interfere. While these estimates have large uncertainty, it is conceivable that the rate of sweeps is limited by recombination [41].

Selection coefficients in HIV have been estimated before by Liu et al. [11] in a patient infected with two HIV-1 subtype B viruses. In this patient, a small number of recombinant forms competed against the ancestral strains and selection differentials were estimated to lie between 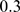
% and 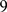
%. These selection coefficients are higher than our estimates, which is plausible since they are associated with entire recombinant genotypes that differ at many sites rather than the single site estimate presented here [39]. The strength of selection for novel epitopes in several HIV genes (rate of escape from cytotoxic T-lymphocyte mediated killing) during the asymptomatic phase of HIV infection has been shown to be of similar magnitude as our estimates [42].

The present study is limited by low time resolution and small sequence samples and more accurate and detailed answers could be obtained from larger samples. Larger sample sizes will require generalizations of the method used to estimate recombination rates which depends on pairs of sites where one of the four possible haplotypes is absent. In large samples, pairs of high frequency polymorphisms will most likely be present in all four possible haplotypes. In this case, one can measure linkage disequilibrium 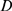 directly and observe how it decays from one timepoint to the next, e.g. by measuring its autocorrelation function 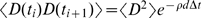. The method presented here is an approximation of this more general method suiteable for noisy data.

## Methods

### Longitudinal sequence data and alignment

Sequences of the C2-V5 region of *env* from 11 patients, which were part of the MACS study [3],[22], were obtained from the Los Alamos National Lab HIV data base (Special interest alignments, accession numbers: AF137629-AF138703, AF204402-AF204670, AY449806 - AY450055 & AY450056 - AY450257). The sequences were translated into amino acid sequences and aligned for each patient separately using MUSCLE v3.6 with default settings [43]. Aligned nucleotide sequences were then constructed by inserting a 3bp gap for each gap in the amino acid alignment. Scripting and plotting was done in Python using the NumPy and Matplotlib environment [44],[45].

### Estimating the recombination rate

To estimate recombination rates, we calculated the frequency of generating the missing haplotype as a function of the distance between polymorphic sites. Specifically, the algorithm proceeds as follows: For each pair of biallelic and gapless sites, we constructed a list of haplotypes, i.e. a list of alleles that co-occur in the same sequence. The list typically contains 3 or 4 haplotypes, but can also contain only 2 of the 4 possible pairs due to undersampling or selection against some of the allele combinations. Only those pairs with 3 haplotypes were included in the estimation. Furthermore, we used only those sites where both alleles were observed at least three times, since rare alleles are very sensitive to sampling noise. The analysis was repeated with this cutoff at two or four alleles, yielding similar results.

For each pair of biallelic sites for which 3 haplotypes were detected in the sample at time 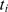, we determined the 4th haplotype that can be formed from the alleles observed at time 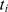, i.e. C…G in the example given in Figure 1. We then checked whether this missing haplotype is detected at time 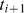. By averaging over all time points (but the last one), all patients, and all pairs within a given distance interval, we determined the frequency of finding the missing haplotype as a function of the distance between the sites and as a function of the product of distance and time difference. The error bars indicated in Figure 2 are calculated as the product of the estimated value and 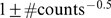. This would be approximately 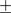 one standard deviation if all counts were independent, which they are not since the observations are pairs of sites and each site contributes to multiple pairs. However, these error bars indicate the relative uncertainties of the different points, which is all that is needed for an unbiased fit. According to Eq. 2, we can estimate the recombination rate from the axis intercept and the slope at 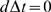, which are extracted from the data by fitting
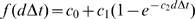
(5)to the data. The fit is done by minimizing the squared deviation, weighted by the relative uncertainty of the data points indicated by the error bars in Figure 2. 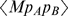 was averaged over all pairs of sites contributing. To estimate a confidence interval for the estimate of 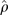, the estimation was repeated 500 times with a set of 11 patients sampled with replacement from the original 11 patients.

The method relies on pairs of biallelic sites in a sample of size 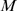, where 3 out of the 4 possible haplotypes are observed. In large samples from a recombining population, one would naively expect to observe all possible haplotypes in most cases. However, due to the very skewed allele frequency distributions 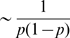, the haplotype formed by the rare alleles is often sufficiently rare that it is missed even in large samples. We denote the alleles at site 1 by 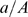 and at site 2 by 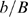, with the 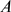 and 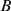 being the minor alleles. First, observe that the mean frequency of haplotype 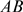, 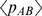, at linkage equilibrium is 
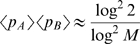
. Hence 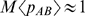 for 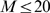, in accord with 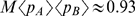 observed in the data. We further assumed that the frequency of the unobserved haplotype, 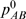, is significantly smaller than 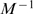. There are several reasons why 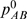 is typically significantly smaller than 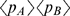 and hence smaller than 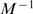: (i) the condition that haplotype 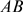 is not observed pushes 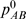 down, (ii) minor alleles tend to be in negative linkage equilibrium if they are involved in selective sweeps, (iii) allele frequency spectra are even more skewed than 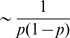 due to purifying selection. The degree to which these 3 reasons reduce 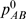 can be best observed in Figure 2B at small 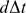. The Figure shows the probability to observe haplotype 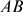 at time 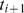, given it was not observed at time 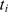. At small 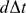, linkage disequilibrium is not yet broken down by recombination and the probability to observe haplotype 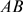 at time 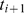 gives us a measure of 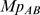 at time 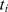, which is indeed much smaller than 1. Hence we can expand 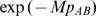 for small 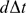 in Eq. 2.

### Estimating the distribution of selection coefficients

The distribution of the rate of change of allele frequencies was estimated from pairs of successive time slices with the following characteristics: (i) site biallelic without indels at time 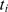 with no constraint on timeslice 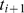, and (ii) monomorphic sites at time 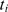 that are biallelic at time 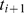 (without indels). In both cases there are two alleles 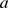 and 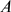 whose frequencies can be meaningfully compared. The difference in allele frequencies were calculated as 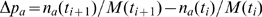, where 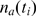 is the count of allele 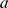 at time 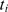 and 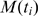 is the sample size. Unless a third allele arose between 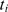 and 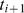 (case (i)), 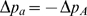 and the common estimate of the rate of change, 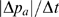, was added to the cumulative distribution. In rare cases where a third allele did arise, both 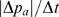 and 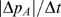 have been added. Sometimes, a nucleotide changed from one state to another between time 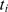 or 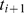 with no polymorphisms detected. In such a case, 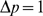 was used.

To detect the action of selection, we produced cumulative histograms of the rate of change in allele frequency for nonsynonymous and synonymous (putatively neutral) polymorphisms by averaging over all patients and all pairs of consecutive time points 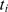 and 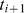 with both 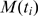 and 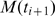 greater than 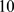. We then tested for excess of fast changes among the nonsynonymous polymorphisms using the Kolmogorov-Smirnov test. The test statistic is the maximal vertical difference between the cumulative distributions 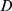 divided by 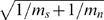, where 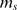 and 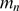 are the number of observations of synonymous and non-synonymous polymorphims. The Kolmogorov-Smirnov test was performed using the statistics module of SciPy [46].

To assess the role of hitch-hiking of synonymous polymorphisms with nearby selected non-synonymous polymorphisms, we produced a list of positions of non-synonymous polymorphisms whose allele freqency changes faster than 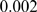 per generation between time 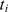 and 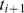. Synonymous polymorphism closer than 100bp to any of these positions where excluded from the conditioned histogram.
